# Comparison of Droplet Digital PCR and Seminested Real-Time PCR for Quantification of Cell-Associated HIV-1 RNA

**DOI:** 10.1371/journal.pone.0085999

**Published:** 2014-01-21

**Authors:** Maja Kiselinova, Alexander O. Pasternak, Ward De Spiegelaere, Dirk Vogelaers, Ben Berkhout, Linos Vandekerckhove

**Affiliations:** 1 HIV Translational Research Unit (HTRU), Department of Internal Medicine, Ghent University and Ghent University Hospital, Ghent, Belgium; 2 Department of Medical Microbiology, Laboratory of Experimental Virology, Center for Infection and Immunity Amsterdam (CINIMA), Academic Medical Center, University of Amsterdam, Amsterdam, The Netherlands; University of British Columbia, Canada

## Abstract

Cell-associated (CA) HIV-1 RNA is considered a potential marker for assessment of viral reservoir dynamics and antiretroviral therapy (ART) response in HIV-infected patients. Recent studies employed sensitive seminested real-time quantitative (q)PCR to quantify CA HIV-1 RNA. Digital PCR has been recently described as an alternative PCR-based technique for absolute quantification with higher accuracy compared to qPCR. Here, a comparison was made between the droplet digital PCR (ddPCR) and the seminested qPCR for quantification of unspliced (us) and multiply spliced (ms) CA HIV-1 RNA. Synthetic RNA standards and CA HIV-1 RNA from infected patients on and off ART (N = 34) were quantified with both methods. Correlations were observed between the methods both for serially diluted synthetic standards (usRNA: R^2^ = 0.97, msRNA: R^2^ = 0.92) and patient-derived samples (usRNA: R^2^ = 0.51, msRNA: R^2^ = 0.87). Seminested qPCR showed better quantitative linearity, accuracy and sensitivity in the quantification of synthetic standards than ddPCR, especially in the lower quantification ranges. Both methods demonstrated equally high detection rate of usRNA in patient samples on and off ART (91%), whereas ddPCR detected msRNA in larger proportion of samples from ART-treated patients (p = 0.13). We observed an average agreement between the methods for usRNA quantification in patient samples, albeit with a large standard deviation (bias = 0.05±0.75 log_10_). However, a bias of 0.94±0.36 log_10_ was observed for msRNA. No-template controls were consistently negative in the seminested qPCR, but yielded a positive ddPCR signal for some wells. Therefore, the false positive signals may have affected the detection power of ddPCR in this study. Digital PCR is promising for HIV nucleic acid quantification, but the false positive signals need further attention. Quantitative assays for CA HIV RNA have the potential to improve monitoring of patients on ART and to be used in clinical studies aimed at HIV eradication, but should be cross-validated by multiple laboratories prior to wider use.

## Introduction

Current antiretroviral therapy (ART) effectively suppresses HIV-1 plasma viremia by inhibiting viral replication. In most patients, plasma viremia is suppressed below the detection limit (20–40 viral copies/ml of plasma) of currently available diagnostic assays [1]. However, even in the settings of optimal therapy, residual low-level viremia persists in a large subset of patients [1], [2]. Because monitoring of ultra-low plasma viremia is technically challenging, and it is unclear whether the low-level viremia has an impact on long-term therapy response, new diagnostic markers and tools will be needed to support HIV care and clinical guidance with the next generation of antiretroviral therapies [1], [3].

Recently, cell-associated (CA) HIV-1 RNA was demonstrated to be a predictive marker of ART outcome in 26 patients [4]. Additionally, CA HIV-1 RNA was found to denote productive HIV-1 infection in patients after therapy cessation and in patients with modest nonadherence to ART [5], [6]. Importantly, as expression of CA HIV-1 RNA is believed to directly reflect the reactivation of latent HIV reservoir *in vivo*, it was recently used to monitor clinical trials aiming to purge the latent reservoir [7], [8]. The role of CA HIV-1 RNA and its potential use as a virological biomarker for monitoring the response to ART and to novel therapeutic strategies has recently been reviewed in depth elsewhere [9]. With the current effort to find a strategy for HIV eradication, an easy and straightforward assay to assess therapy effectiveness is needed. In this framework, CA HIV-1 RNA is a promising candidate biomarker for future diagnostic purposes.

Despite promising data indicating the importance of monitoring CA HIV-1 RNA load in patients on ART, only a limited number of studies have been conducted on these markers. This is mainly due to the technical difficulties to monitor the low amounts of HIV-1 RNA. In recent years, quantification of CA HIV-1 RNA has been performed using assays based on quantitative reverse transcription real-time PCR (RT-qPCR) [5], [7], [8], [10]. However, this technique suffers from increased technical variation at the lower ranges of detection [11]. Moreover, small differences in efficiency in the lower ranges of the standard curve may further bias quantitative results [12], [13]. To overcome these shortcomings, Pasternak et al. developed a seminested real-time qPCR procedure that enables CA HIV-1 RNA measurement in patient samples with a lower limit of quantification and with increased accuracy at the lower quantitative range compared to one-step qPCR based assays [14]. By performing two successive PCR reactions, the specificity is maintained and the limit of quantification is considerably reduced. The introduction of this method revealed its value in multiple *in vivo* studies [4], [6], [14], [15]. However, an accurate standard curve is still necessary for seminested qPCR quantification. This requires careful calibration and assumes consistent amplification efficiencies between the biological samples and the standards. A quantitative technique that does not rely on a standard curve is therefore desirable.

Digital PCR (dPCR) has been described as an alternative PCR-based technique for absolute quantification with higher accuracy compared to qPCR [12], [13], [16]. The dPCR technique is based on limiting dilution of samples across a large number of separate PCR reactions. If the input sample is sufficiently diluted, not all reactions will harbor template DNA. This will allow absolute quantification using Poisson statistics without requiring a standard curve [12], [17]. In addition, decreased PCR efficiency is better tolerated in dPCR as the end-point fluorescence suffices to perform absolute quantification. Because of technical obstacles and costs of making multiple reactions, dPCR has not been widely implemented so far. However, thanks to recent technological developments including microfluidics to form droplet in oil suspension, dPCR is now possible in high throughput at lower costs. To date, several studies on cancer and viral infections report a higher degree of sensitivity and precision of dPCR than qPCR [13], [16], [18], [19]. In addition, Henrich et al. reported equal sensitivity of ddPCR and qPCR for detection of HIV-1 DNA in patient samples [20]. On the other hand, one study evaluated CMV viral load quantification and reported reduced sensitivity of dPCR compared to qPCR [21]. Taken together, the published data point to the potential clinical use of dPCR for sensitive and accurate absolute quantification of nucleic acids.

In this study, we compared seminested qPCR and digital droplet PCR (ddPCR) for quantification of CA HIV RNA. We first quantified the synthetic RNA standards, corresponding to unspliced (us) and multiply spliced (ms) CA HIV-1 RNA, by ddPCR and seminested qPCR. Based on the quantification of these standards, raw data-to-RNA conversion factors were generated for both methods. These conversion factors were subsequently used, in the patient samples, to convert the raw outputs of seminested qPCR (quantification cycles, Cq) and ddPCR (cDNA copy numbers) to the HIV RNA copy numbers. This allowed making a comparison between ddPCR and the seminested qPCR for quantification of CA HIV-1 RNA in the samples from HIV-infected patients on and off ART.

## Materials and Methods

### Patient Material

Thirty-four peripheral blood mononuclear cell (PBMC) samples used in this study were from patients who were participating in the Primo-SHM cohort at the Academic Medical Center (AMC) in Amsterdam, the Netherlands (n = 23) and patients who are in follow-up at the Aids Reference Center of Ghent University Hospital (n = 11). Samples were collected from patients receiving ART with undetectable viral loads (<50 copies/ml) (n = 14 in Amsterdam cohort and n = 7 in Ghent cohort), and from therapy-naïve patients (n = 9 in Amsterdam cohort and n = 4 in Ghent cohort). The majority of patient samples (28 out of 34) were derived from patients infected with HIV-1 subtype B, two samples were subtype CRF01_AE, one was subtype CRFO2_AG, and for three samples the subtype was unknown (Table S1). Ethical approval was obtained from Ethics Committees of the University Hospital Ghent and of the AMC. All participants had provided written informed consent.

### Nucleic Acid Isolation, DNase Treatment and cDNA Synthesis

Cell-associated HIV-1 RNA from patient samples of Ghent University Cohort was extracted from 5×10^6^ PBMCs using TRIzol® Reagent (Ambion®) and eluted in 20 µl nuclease-free water (Ambion®) as previously described [22]. RNA purity and integrity was assessed using automated electrophoresis system (Experion™, Bio-Rad) [22]. Total cell-associated nucleic acids from patient samples of the Amsterdam cohort were extracted from 2.5–5×10^6^ PBMCs according to the isolation method of Boom et al. and eluted in 50 µl nuclease-free water (Ambion®) [23]. 12 µl of the eluted RNA samples were first subjected to DNase treatment (DNA-free™ kit, Ambion®), to remove HIV-1 DNA which could interfere with the quantification, and subsequently added to the reverse transcription (RT) mix. RT was performed in the total volume of 20 µl reaction and contained 200 units of SuperScript™ III reverse transcriptase, 20 units of RNaseOUT™ ribonuclease inhibitor, 150 ng of random primers, and 20 nmoles of deoxynucleoside triphosphates (all- Invitrogen) at 42°C for 60 min, followed by heat inactivation of the reverse transcriptase for 10 min at 70°C. Patient-derived cDNA preparations were used for the usRNA and the msRNA assays by ddPCR and seminested qPCR. For all samples, same amounts (4 µl) of the same cDNA preparations (hereafter referred to as “input unit”) were always used for both ddPCR and qPCR, except for 11 patient samples with limited amounts of material, where 1 µl of cDNA template was used for the seminested qPCR and the results were normalized to 4 µl for the purpose of subsequent comparisons. Samples were tested in single replicate, because of the limited availability of patient samples (Graph S1).

### No-template Controls

For both usRNA and msRNA assays and for both ddPCR and qPCR methods, no-template controls (NTCs) with water were included in every run. To assess possible false positive droplets for the ddPCR run, a total of 42 NTCs were assessed. From these, 21 NTCs were assessed for the usRNA assay and 21 for the msRNA assay. To discern possible PCR contamination from system artefacts, eight NTCs per assay were prepared with an amplification-deficient ddPCR mix, which contained only one primer (only forward or only reverse) and a probe. These eight wells were surrounded with other NTCs. Additionally, to assess the possibility of cross-contamination between wells in the droplet read-out, 4 positive controls were inserted between the NTC wells. The read-out of the dPCR proceeds in a sequential manner, hence the 4 positive controls were placed in the wells just before the NTCs.

### Primers and Probes

For both PCR methods, previously described primer and probe sets for usRNA and msRNA were used [5], [14]. A table that lists all primers and probes is provided as supplementary data (Table S2). HIV-1 usRNA was quantified using the GAG1, GAG2, and SK431 primers that amplify a region within the HIV-1 *gag*, and the GAG3 hydrolysis probe was used. Spliced HIV-1 msRNA was quantified using the ks1, mf84, and mf83 primers that amplify a region containing the *tat/rev* exon-exon junction, and the ks2-tq hydrolysis probe.

### Droplet Digital PCR

HIV-1 RNA was quantified using the QX100™ Droplet Digital™ PCR system (Bio-Rad, Pleasanton, CA). The ddPCR mix for the usRNA assay consisted of: 10 µl 2x ddPCR™ super mix for probes (Bio-Rad); 200 nM of GAG1 and GAG2 primers; 400 nM GAG3 probe mix and 4 µl of the cDNA into a final volume of 20 µl. The total mix was placed into the 8 channel cartridge, 50 µl of droplet generating oil was added and droplets were formed in the QX100™ droplet generator (Bio-Rad). Droplet in oil suspensions were transferred to an Eppendorf® 96 well plate (Eppendorf, Germany) and placed into the T100™ Thermal Cycler (Bio-Rad). Cycling conditions were as follows: 95°C for 5 min, followed by 40 cycles of 95°C for 15 sec and 58°C for 60 sec. The ddPCR mix for the msRNA assay consisted of 10 µl 2x ddPCR™ super mix for probes (Bio-Rad); 250 nM of ks1 and mf83 primers, 500 nM of ks2-tq, and 4 µl of the cDNA into a final volume of 20 µl. PCR cycling conditions were the same as for the usRNA assay, except the annealing temperature which was 60°C. The droplets were subsequently read automatically by the QX100™ droplet reader (Bio-Rad) and the data was analyzed with the QuantaSoft™ analysis software 1.3.2.0 (Bio-Rad).

### Seminested Real Time PCR

For the seminested qPCR, two rounds of PCR amplification were performed for usRNA and msRNA assays. For the usRNA assay, the first round of the PCR was performed on a conventional PCR machine (GeneAmp® PCR System 9700; Applied Biosystems®) in 25 µl of PCR mix, which contained 4 µl of cDNA template, 20 mM Tris (pH 8.3), 50 mM KCl, 2 mM MgCl2, 0.4 mM of deoxynucleoside triphosphates, 1 U of AmpliTaq® (Applied Biosystems®), and 50 ng each of GAG1 and SK431 primers. The PCR cycling settings were: 94°C for 3 min, followed by 15 cycles of 94°C for 30 s, 55°C for 30 s, and 72°C for 1 min. The product of the first PCR was used as a template in the second, seminested qPCR amplification, performed on the ABI Prism® 7000 qPCR machine (Applied Biosystems®) using TaqMan® detection chemistry. Two microliters of the first PCR product were diluted to 50 µl with PCR mix containing 25 µl of 2*Platinum® Tag qPCR mix (Invitrogen), 1 µl of ROX reference dye (Invitrogen), 1 mM MgCl2, 0.2 µM of each of primers (GAG1 and GAG2), and 0.2 µM dual hybridization probe GAG3. Real-time PCR cycling settings were: 50°C for 2 min, 95°C for 10 min, 45 cycles of 95°C for 15 s and 60°C for 1 min. For the msRNA assay, the same protocol was used. The first PCR was performed with the primer pair ks1 and mf83, which amplifies the msRNA species encoding the Tat and Rev proteins. Subsequently, the seminested qPCR of the msRNA assay was performed with the primers mf84 and mf83 and the fluorescent hydrolysis probe ks2-tq. Cycling conditions were the same, except that 50 amplification cycles were done instead of 45 in the second PCR.

### Preparation of Standard Curves

As external standards, synthetic runoff RNA transcripts, corresponding to the HIV *gag* and *tat/rev* regions, were used [14]. The concentrations of RNA standards were determined spectrophotometrically and recalculated to RNA copies/µl. Master stocks of the standards were frozen in aliquots at −80°C until use. Duplicate standard curves for each assay were made from separate master stocks from which serial dilutions were made to obtain a 7-point standard curve. Serial dilutions of standards for usRNA and msRNA assays were quantified using the ddPCR and the seminested qPCR technique.

### Conversion of the Raw Data

**Figure 1:**
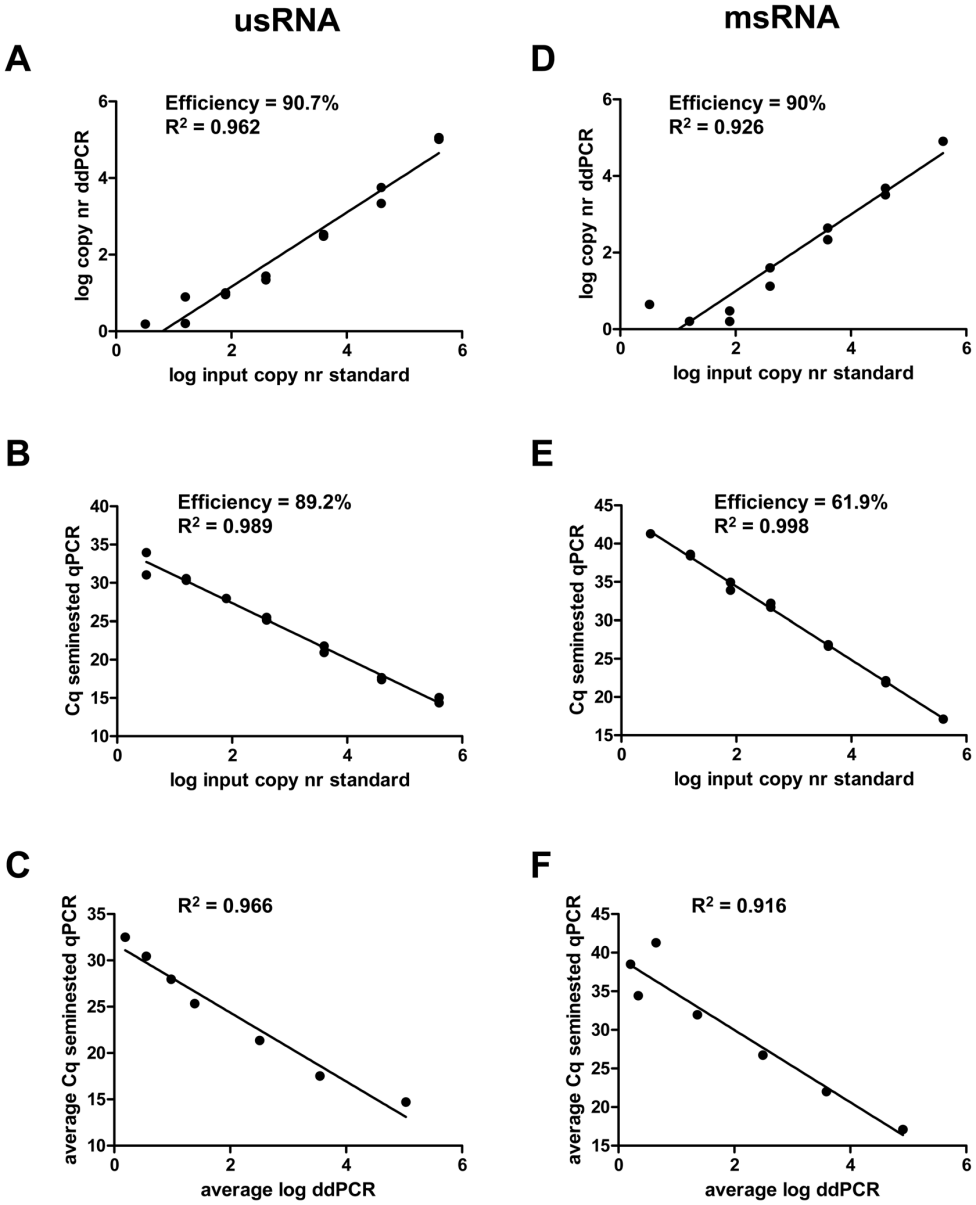
Quantification of serially diluted synthetic RNA standards by ddPCR and seminested qPCR. Panels (A) – (C) show usRNA data, and panels (D) – (F) show msRNA data. (A, D) Quantification of standards by ddPCR. The log_10_-transfomed RNA copy numbers of serially diluted synthetic RNA standards were plotted against the corresponding log_10_-transformed cDNA copy numbers determined by ddPCR and fitted with a linear regression model. (B, E) Quantification of standards by seminested qPCR. The log_10_-transfomed RNA copy numbers of serially diluted synthetic RNA standards were plotted against the corresponding quantification cycle (Cq) values of seminested qPCR on a semi-log scale and fitted with a linear regression model. (C, F) Pearson correlation between ddPCR and seminested qPCR output values for the serially diluted standards. The log_10_-transformed cDNA copy numbers determined by ddPCR were plotted against the corresponding quantification cycle (Cq) values of seminested qPCR on a semi-log scale. For every dilution, an average value of two independent measurements is shown.

The raw quantitative output of ddPCR was the cDNA copy number in the input sample, whereas qPCR provided the Cq value which is based on the fluorescent amplification curve. For patient samples, the raw outputs of both methods were converted to the RNA copy numbers using the standard curves as conversion factors (Table 1, Fig. 1) [14]. The quantified HIV RNA copy numbers were log-transformed. The final output measures, for patient samples, were the log_10_ RNA copy numbers per input unit (4 µl of the input cDNA) for both ddPCR and qPCR (Graph S1).

**Table 1:** Quantification of standards for usRNA and msRNA with ddPCR and seminested qPCR.

	usRNA				msRNA			
	ddPCR		Seminested qPCR		ddPCR		Seminested qPCR	
	Mean ± SD[i]	CV[ii] (%)	Mean ± SD	CV (%)	Mean ± SD	CV (%)	Mean ± SD	CV (%)
Copy nr. ofstandard	(log_10_ cDNAcopies/reaction)		(cq[iii])		(log_10_ cDNAcopies/reaction)		(Cq)	
4×10^5^	5.03±0.03	0.7	14.71±0.49	3.3	4.91±0.00	0.1	17.12±0.01	0.0
4×10^4^	3.55±0.29	8.3	17.53±0.21	1.2	3.59±0.12	3.3	22.01±0.19	0.9
4×10^3^	2.51±0.04	1.5	21.37±0.60	2.8	2.49±0.21	8.5	26.74±0.15	0.6
4×10^2^	1.39±0.07	5.2	25.34±0.21	0.8	1.36±0.34	25.1	31.95±0.37	1.2
8×10^1^	0.98±0.03	2.6	27.97±0.01	0.0	0.34±0.20	57.9	34.44±0.73	2.1
1.6×10^1^	0.55±0.50	90.5	30.45±0.17	0.6	0.21	n/a	38.51±0.15	0.4
3.2×10^0^	0.09	n/a	32.50±2.05	6.3	0.65	n/a	41.30	n/a

[i] SD, standard deviation.

[ii] CV, coefficient of variation.

[iii] Cq, quantification cycle.

### Statistical Analysis

Statistical analysis was performed using GraphPad Prism software 5.01 (http://www.graphpad.com). Linear regression was used to analyze the standard curves. Pearson correlation analysis and Bland-Altman tests were used to assess the quantitative agreement between ddPCR and seminested qPCR measurements in patient samples. For these analyses, undetectable values were censored to one copy. Fisher’s exact tests were used to compare the detectability of HIV RNA in patient samples between the methods.

## Results

### Detection of HIV-1 RNA in Standards

Serially diluted usRNA and msRNA standards were measured in duplicate for both ddPCR and seminested qPCR methods. Table 1 shows the raw quantification output of serially diluted synthetic RNA standards by ddPCR (cDNA copy numbers) and seminested qPCR (Cq values). The quantitative accuracy was higher for seminested qPCR as compared with ddPCR, as the coefficients of variation (CV) were smaller for seminested PCR for almost all standard dilutions, especially in the lower ranges of quantification. The detectability of higher copy numbers of standards was the same between the two methods, but for the lower concentrations, seminested qPCR showed better sensitivity than ddPCR for both usRNA and msRNA standards (Table 1).

Quantitative linearity of both methods was assessed by quantification of standards for usRNA and msRNA. UsRNA standard curve showed good efficiency (E) and linearity i.e. E = 90.7%, R^2^ = 0.96 by ddPCR and E = 89.2%, R^2^ = 0.99 by seminested qPCR, respectively (Fig. 1A and 1B). The correlation between ddPCR and seminested qPCR on standards had a coefficient of determination R^2^ of 0.97 (Fig. 1C). Linearity of standard curve for msRNA was better in seminested qPCR: R^2^ = 0.93 in ddPCR and R^2^ = 0.998 in seminested qPCR. However, PCR efficiency was better in ddPCR (90.0%) than in seminested qPCR (61.9%) (Fig. 1D and 1E). The correlation between the two methods had a coefficient of determination R^2^ of 0.92 (Fig. 1F).

### Detection of HIV-1 RNA in Patient Samples

Thirty-four clinical samples were evaluated, with 21 samples from patients on ART with undetectable viral load (<50 copies/ml) and 13 samples from therapy-naïve patients. UsRNA and msRNA quantification was performed with both methods. UsRNA was quantified in all 34 patient samples and msRNA was quantified in 23 samples (15 from patients on ART and 8 from therapy naïve patients).

The detectability of usRNA in patient samples was equally high for both methods: ddPCR and seminested qPCR detected usRNA in 31 out of 34 samples (91%) (Table S1). From therapy-naïve patients, both methods detected usRNA in 12 out of 13 samples (92%). From patients on ART, usRNA was detected in 19 out of 21 samples (90%) by both methods. MsRNA was detected more frequently with the ddPCR (14 positive samples out of 23, 61%) than seminested qPCR (9 positive samples out of 23, 39%) (*p* = 0.24). This difference is attributable to samples from patients on ART: whereas the detectability of msRNA in therapy-naïve patients was equal between methods (6 out of 8 samples (75%) were positive by both methods), msRNA was detected in 8 out of 15 samples from ART-treated patients (53%) by ddPCR and in 3 out of 15 such samples (20%) by seminested qPCR (*p* = 0.13). Detection of usRNA and msRNA with both methods in patient samples on and off ART is listed in the supplementary table S1.

**Figure 2:**
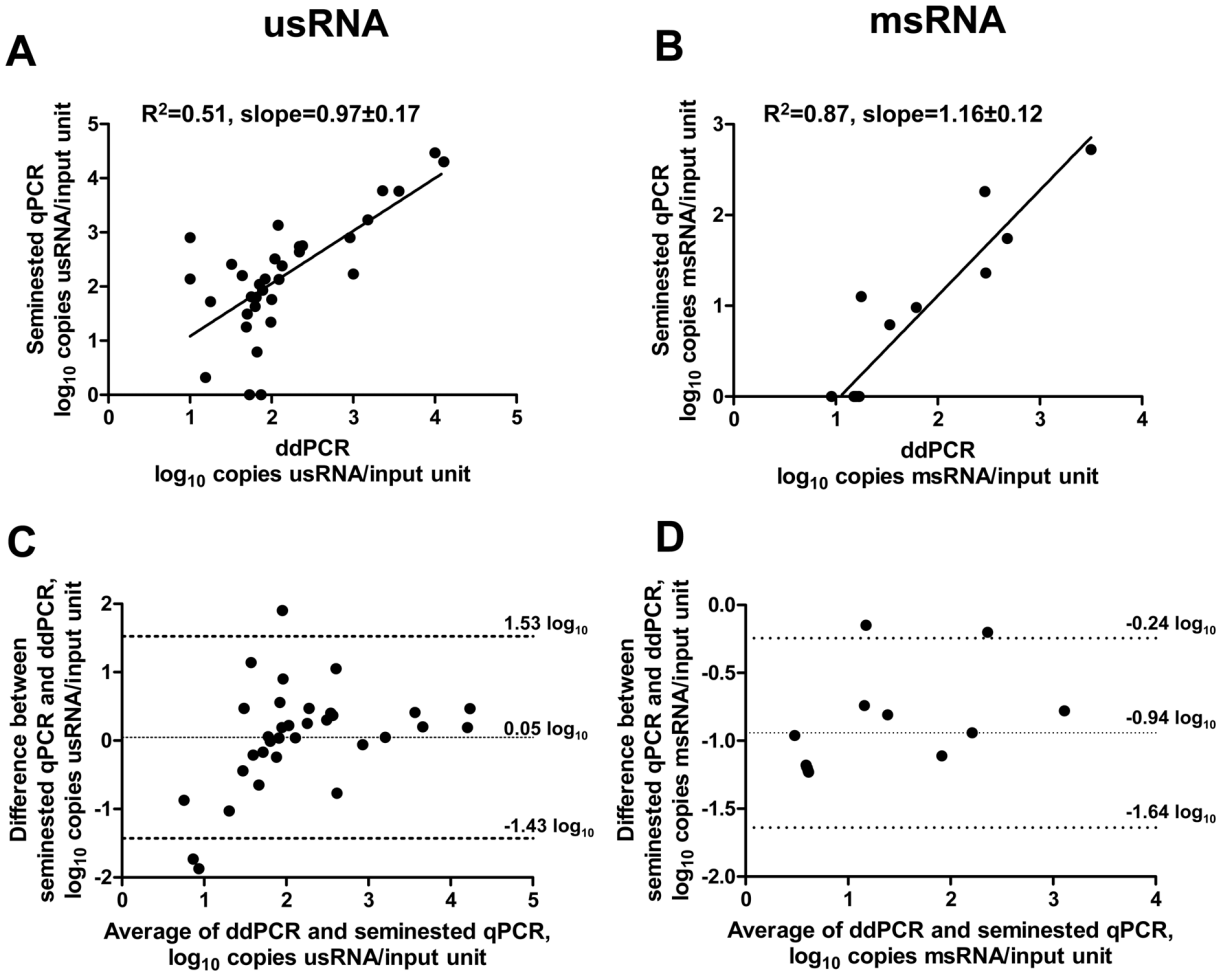
Quantification of usRNA and msRNA in patient samples. (A, B) Correlations between ddPCR and seminested qPCR measurements of usRNA (A) and msRNA (B) in patient samples are shown. The units of measurement are log_10_ copies RNA per input unit (4 µl of input cDNA) for both ddPCR and qPCR. Samples that were undetectable with both methods (n = 1 for usRNA and n = 8 for msRNA) are not shown. (C, D) Bland-Altman plots comparing the ddPCR and seminested qPCR measurements of usRNA (C) and msRNA (D) in patient samples. Mean differences and 95% Limits of Agreement are shown on the graphs.

Analysis of patient samples by ddPCR and seminested qPCR revealed a correlation between the two methods (R^2^ = 0.51 for usRNA and R^2^ = 0.87 for msRNA; Fig. 2A and 2B). For usRNA, the mean difference (bias) between the RNA copy numbers generated with seminested qPCR and ddPCR, assessed with Bland-Altman test, was 0.05±0.75 log_10_ (95% Limits of Agreement, −1.43 to 1.53 log_10_) and a corresponding bias for msRNA was −0.94±0.36 log_10_ (95% Limits of Agreement, −1.64 to −0.24 log_10_; Fig. 2C and 2D).

No-template controls (NTCs) were used in all assays for both methods. In the seminested qPCR protocol, all NTC remained negative in the usRNA and msRNA assays. However, in ddPCR, positive events of 0.16 copies/reaction (2 positive droplets) and 0.22 copies/reaction (3 positive droplets) were detected in 1 well out of 3 for the usRNA and msRNA assay, respectively. The positive NTC in the usRNA assay had 3 droplets with similar fluorescence range as for the patient samples (Fig. S1). The positive NTC in msRNA had 2 droplets with higher fluorescence than the patient samples (Fig. S2).

To better understand the nature of false-positive events that were observed, we assessed another 42 NTC replicates. From these NTCs, 1 or 2 positive droplets were registered in 9 wells out of the total 42. From these, only 1 well with 1 positive droplet originated from the usRNA assay, and the remaining 8 wells were from the msRNA assay. Two wells from the msRNA assay had 2 positive droplets detected and in the remaining 6 wells, only 1 positive droplet was registered. Interestingly, the reactions in the wells with 2 positive droplets and in 1 well with 1 positive droplet were prepared with the amplification-deficient ddPCR mix. The 4 out of 42 NTCs that were placed after a positive control with high input of amplicons were all negative.

## Discussion

In the present study, synthetic HIV RNA standards and CA HIV RNA in patient-derived samples were quantified with seminested qPCR and ddPCR. To the best of our knowledge, this is the first report of HIV RNA measurement by ddPCR in patient-derived samples. In the first part of the study, synthetic HIV RNA standards were measured by both seminested qPCR and ddPCR. Subsequently, in the second part, patient-derived samples were quantified with both methods.

The correlation of measurements between seminested qPCR and ddPCR was good for both standard curves (Figs. 1C, 1F). However, in absolute numbers, the cDNA copy numbers quantified by ddPCR were lower than the corresponding RNA copy numbers assessed by UV spectrophotometry. One explanation for this is the suboptimal efficiency of RT, in which not all RNA molecules are reverse transcribed into cDNA. The efficiency of RT (cDNA yield) has been shown to vary widely, depending on the enzyme used and the priming strategy [24], [25]. Another possible explanation for the discrepancy between the RNA and cDNA copy numbers is molecular dropout [26], a recently described dPCR phenomenon, in which the target molecule is present in the partition but fails to amplify. Because we directly used aliquots of RT reactions as input material for ddPCR, the PCR step could have been inhibited by RT components [27]. To correct for the RT efficiency and PCR inhibition, dPCR quantification of RNA should be performed in combination with a calibrator that would provide a conversion factor for the raw dPCR data to RNA copy numbers [27]. In our study, the raw data-to-RNA conversion factors were calculated based on the standard curves. These conversion factors were used for the patient samples to convert the Cq to RNA copy numbers for the seminested qPCR, and the cDNA copy numbers to RNA copy numbers for the ddPCR.

The second part of the study consisted of quantification of patient-derived material with both seminested qPCR and ddPCR. Thus far, there are no clinically validated methods for measurement of CA HIV RNA. However, the seminested qPCR method has been validated and compared with Cobas Amplicor HIV-1, the clinical assay for plasma HIV RNA quantification [14]. Moreover, the seminested qPCR method has been extensively used for CA HIV RNA quantification in patient-derived material [4], [6], [14], [15]. Hence, we compared seminested qPCR and ddPCR for CA HIV RNA measurements in patient samples.

For usRNA quantification in patient samples, the difference between seminested qPCR and ddPCR was 0.05±0.75 log_10_ (Fig. 2C). On average, it is less than the accepted threshold of clinically significant variability (0.5 log_10_). However, the standard deviation was relatively large and the linearity of the correlation was only R^2^ = 0.51 (Fig. 2A). The suboptimal correlation could be due to the fact that the majority of samples tested were derived from well suppressed patients on ART, in whom usRNA levels are very low. It is well known that in samples with very low copy numbers, random variation due to sampling error (Poisson’s error) becomes significant [28], [29]. This indicates that the comparison of methods on samples from patients on suppressive ART is difficult. Consequently, a second assessment was made, comparing only those patient samples with more than 100 copies/input unit detected with both methods. This resulted in an improved correlation, R^2^ = 0.87 (data not shown) supporting the hypothesis that the mediocre correlation in the usRNA assay was primarily due to sampling variation in samples with low HIV-1 loads.

For msRNA quantification in patient samples, we observed a higher difference between the measurements by seminested qPCR and ddPCR (–0.94±0.36 log_10_), meaning that msRNA values measured by seminested qPCR were lower than the corresponding ddPCR measurements by an average factor of 8.7. The underestimation of measurements with seminested qPCR could be due to primer-template or probe-template mismatches. Such mismatches have a direct effect on qPCR-based quantification [30], but dPCR is less susceptible to these effects [16]. This is supported by a recent comparison of the effects of mismatches on quantifying HIV DNA with qPCR and ddPCR [16].

While the detectability of usRNA in patients on ART and in therapy-naïve patients was equally high (90–92%) with both methods, msRNA was detected with ddPCR in a higher proportion of patients on ART compared to the seminested qPCR. However, this difference did not achieve statistical significance, possibly due to small patient numbers. Moreover, we cannot conclude that this effect is due to higher sensitivity of the ddPCR, because samples containing single positive droplets in ddPCR may have been false positives due to the observed false positive reactions in the ddPCR NTCs. The detectability of msRNA in therapy-naïve patients with higher msRNA loads was equal between the methods.

The major limitation of this study is the positive signals obtained in the NTCs in the ddPCR experiments for both usRNA and msRNA assays. Although the majority of NTCs were negative, several NTCs were recorded with ≤3 positive events. The false positive signals may have contributed to the higher detectability of msRNA in patients on ART by ddPCR. The current ddPCR technique does not allow sequencing of the samples in order to establish whether the false positives represent artefacts. The problem of false positives could be alleviated by setting up a limit of detection for ddPCR, which would correspond to the maximal number of positive droplets per NTC well (3 in this study). In this case, some of the samples of patients on ART that were positive for usRNA, would not be scored as positives, as they yielded ≤3 positive droplets. Likewise, all samples from patients on cART that were positive for msRNA would not be scored as positive because they yielded ≤2 positive droplets (Table S3).

Our study is not the first occasion on which false positive events in NTCs were reported in ddPCR experiments. Previously, two independent groups reported positive signals in NTCs, and Strain et al. reported on average of 0.1 to 0.4 false positive events per NTC well after analyzing more than 500 NTCs [16], [31]. These false positive events are detected randomly, they are not assay-dependent, and they have different fluorescence height (Fig. S1 and S2). Sometimes we observed false positive events with extremely high fluorescence compared to the real positives, suggesting that these are artefacts (Fig. S2). This is supported by the experiments on the NTCs which showed that false positives also occurred in reactions where lab contamination can be excluded. In addition, these experiments revealed that possible carry-over during sample processing and read-out is also unlikely. At the moment, the false negative events, appearing in experiments with ddPCR, preclude its wider use for quantification of extremely low viral loads, and this problem needs to be further dealt with.

Recent interest in ddPCR as a method of nucleic acid quantification largely stems from the fact that ddPCR is a direct method that does not rely on an external standard curve, as qPCR does. However, although ddPCR does provide absolute quantification of target DNA (or cDNA), it is important to realize that, at this point, its application to absolute quantification of RNA is still under development. When using the two-step RT-dPCR method, where RNA is reverse transcribed to cDNA before sample partitioning, the quantified absolute cDNA copy number has to be back-converted to the RNA copy number. In this study, this cDNA-to-RNA conversion was performed based on the standard curve, which enabled the direct comparison of RNA copy numbers in patient samples between the two methods, but made the ddPCR quantification of RNA as relative on the standard curve as the seminested qPCR was. The use of pre-validated calibrators will facilitate higher accuracy of RNA quantification with ddPCR. An alternative is to use one step RT-dPCR methods, in which an RNA sample is partitioned prior to RT. However, accurate calibrators will likely be needed even in this case, because the efficiency of RNA quantification by dPCR was recently shown to be assay- and transcript-dependent [27].

Further exploration of the use of ddPCR for accurate CA HIV RNA measurement is necessary. Quantitative assays for CA HIV RNA have the potential to improve the monitoring of patients on ART and to be used in clinical studies aimed at HIV eradication, but should be cross-validated by multiple laboratories prior to wider use.

## Supporting Information

Figure S1 **Negative template controls for us- and msRNA assays in ddPCR.** DdPCR droplet read-out for the usRNA assay. Last three columns (H03, H04 and H05) show the NTC’s. In column H04 three positive droplets are registered. The other two columns are the other 2 NTC, which are negative. The readout from A01 until G03 is for patient samples.
(TIF)

Figure S2 **DdPCR droplet read-out for the msRNA assay.** The last column (H05) shows the NTC with two positive droplets registered. H03 and H04 columns are the other two NTC, which are negative. The readout from A01 until G03 is for patient samples.
(TIF)

Table S1 **Patient samples information; detection of usRNA and msRNA with ddPCR and seminested qPCR platform.**
(XLSX)

Table S2 **Primers and probes used in this study.**
(XLSX)

Table S3 **Raw data from the ddPCR experiments for usRNA and msRNA assays in patient samples.**
(XLSX)

Graph S1 **Workflow used to measure usRNA and msRNA in clinical samples on ddPCR and seminested qPCR.**
(DOCX)
