# Supplementary figures and images for: Comparison of Droplet Digital PCR and Seminested Real-Time PCR for Quantification of Cell-Associated HIV-1 RNA
Source: PLoS One. 2014 Jan 21;9(1):e85999. doi: 10.1371/journal.pone.0085999 (PMC3897572; doi:10.1371/journal.pone.0085999)

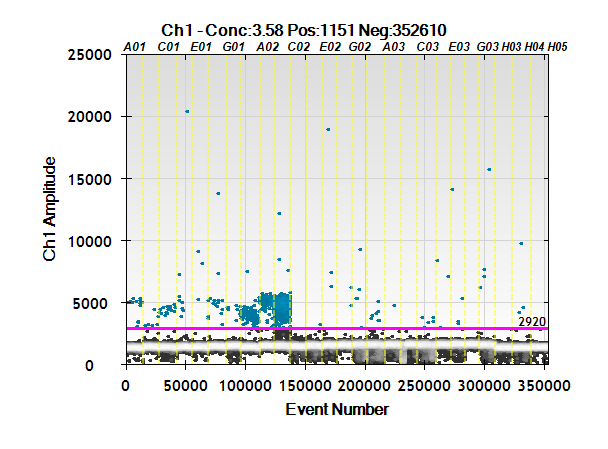

Supplement: Figure S1 — Negative template controls for us- and msRNA assays in ddPCR. DdPCR droplet read-out for the usRNA assay. Last three columns (H03, H04 and H05) show the NTC’s. In column H04 three positive droplets are registered. The other two columns are the other 2 NTC, which are negative. The readout from A01 until G03 is for patient samples. (TIF) [file pone.0085999.s001.tif]

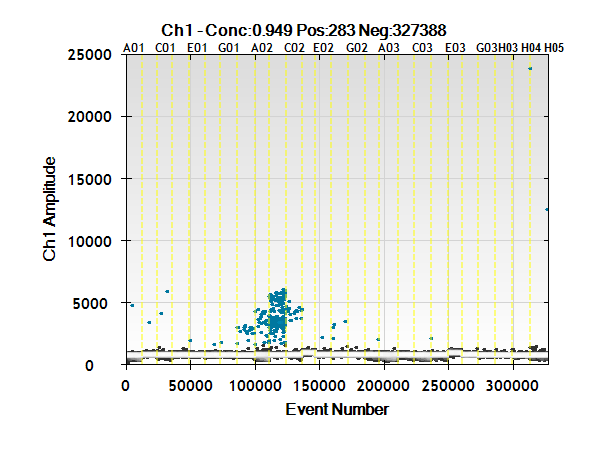

Supplement: Figure S2 — DdPCR droplet read-out for the msRNA assay. The last column (H05) shows the NTC with two positive droplets registered. H03 and H04 columns are the other two NTC, which are negative. The readout from A01 until G03 is for patient samples. (TIF) [file pone.0085999.s002.tif]
