# Supplementary material for: Comparison of Droplet Digital PCR and Seminested Real-Time PCR for Quantification of Cell-Associated HIV-1 RNA
Source: PLoS One. 2014 Jan 21;9(1):e85999. doi: 10.1371/journal.pone.0085999 (PMC3897572; doi:10.1371/journal.pone.0085999)

Graph S1. Workflow used to measure usRNA and msRNA in clinical samples on ddPCR and seminested qPCR


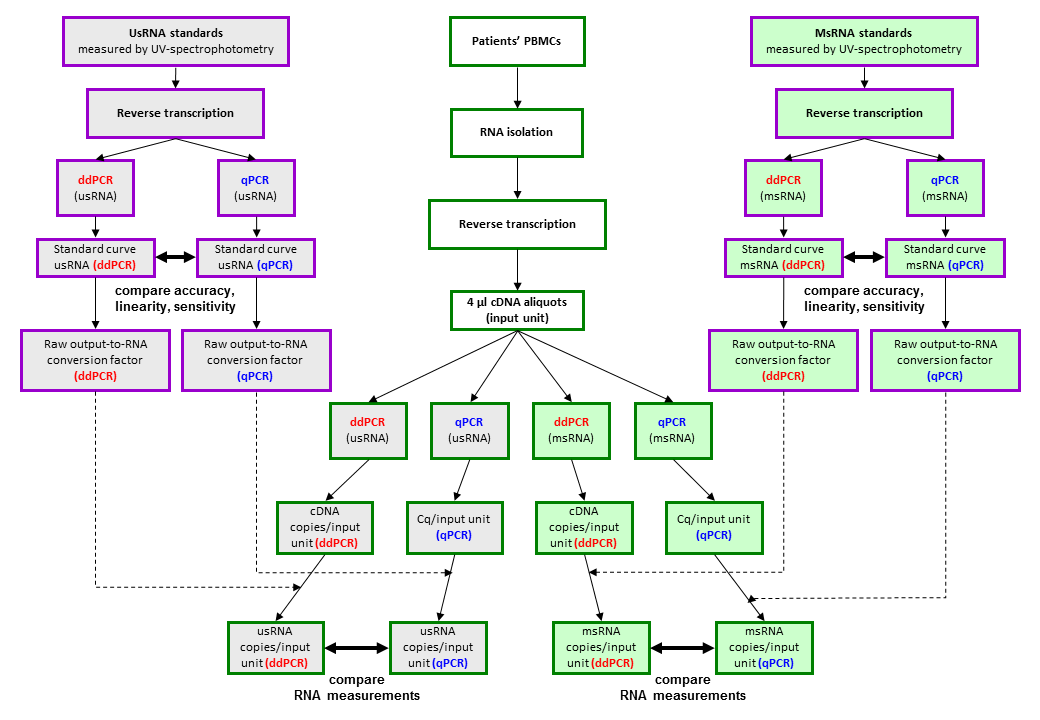

Supplement: Graph S1 — Workflow used to measure usRNA and msRNA in clinical samples on ddPCR and seminested qPCR. (DOCX) [file pone.0085999.s006.docx]
